# Data-Driven Prediction of Vessel Propulsion Power Using Support Vector Regression with Onboard Measurement and Ocean Data

**DOI:** 10.3390/s20061588

**Published:** 2020-03-12

**Authors:** Donghyun Kim, Sangbong Lee, Jihwan Lee

**Affiliations:** 1 Korea Marine Equipment Research Institute, Busan 49111, Korea; kimdonghyun9942@gmail.com; 2 Lab021, Busan 48508, Korea; sblee@lab021.co.kr; 3 Division of Systems Management and Engineering, Pukyong National University, Busan 48513, Korea

**Keywords:** vessel power prediction, data-driven prediction, support vector regression, ISO15016, onboard measurement data, ocean whether data, predictive analytics

## Abstract

The fluctuation of the oil price and the growing requirement to reduce greenhouse gas emissions have forced ship builders and shipping companies to improve the energy efficiency of the vessels. The accurate prediction of the required propulsion power at various operating condition is essential to evaluate the energy-saving potential of a vessel. Currently, a new ship is expected to use the ISO15016 method in estimating added resistance induced by external environmental factors in power prediction. However, since ISO15016 usually assumes static water conditions, it may result in low accuracy when it is applied to various operating conditions. Moreover, it is time consuming to apply the ISO15016 method because it is computationally expensive and requires many input data. To overcome this limitation, we propose a data-driven approach to predict the propulsion power of a vessel. In this study, support vector regression (SVR) is used to learn from big data obtained from onboard measurement and the National Oceanic and Atmospheric Administration (NOAA) database. As a result, we show that our data-driven approach shows superior performance compared to the ISO15016 method if the big data of the solid line are secured.

## 1. Introduction

The fluctuation of the oil price and unstable shipping rates have enforced ship builders and shipping companies to improve the energy efficiency of vessels [1]. Since the fuel cost is the largest portion of the operating cost of a vessel, improving energy efficiency can result in huge savings in the total operating cost [2]. Achieving good energy efficiency is also a prerequisite to cope with demanding environmental regulations because more energy-efficient vessels can reduce fuel consumption and greenhouse gas emissions [3]. Therefore, several technological solutions have been proposed to improve the energy efficiency of vessels [4]. Design optimization technology such as ‘hull form optimization’ [5] or ‘propeller configuration’ [6] try to design vessels to improve the fuel efficiency. On the other hand, operational optimization technologies aim to improve the operational performance by finding optimal speed and optimized voyage routes of vessels [7,8]. For a more comprehensive list of energy saving technologies, the reader is referred to Tilling et al. [4].

To measure the effectiveness of energy-saving technology, it is necessary to measure the speed/power performance of the vessel [9]. Today, many ship owners leave the responsibility of the delivery trials including speed-power trials with the shipyard [10]. However, the speed/power relationship obtained from a testing environment cannot be generalized to realistic operating conditions and the propulsion performance of the vessel may be affected by several external factors such as wind, tide, wave or hull fouling [11]. Thus, in a dynamic environment, it is difficult to determine exactly whether the increase in energy efficiency is due to newly adopted energy-saving solutions or other factors. 

As an internationally recognized standard, ISO15016 is proposed to measure the speed/power relationship from the delivery trials [12]. ISO15016 also introduces several calculation procedures to adjust the impact of external factors on the propulsion performance. Unfortunately, those procedures are computationally expensive because many input parameters are required and their related equations are complex [13]. As an alternative measure, the Energy Efficiency Operation Index (EEOI) [14] was proposed to measure the energy efficiency of the vessel. However, EEOI is just an aggregated index that is obtained by the total amount of fuel usage and cannot be used to predict the relationship between the propulsion performance and other related factors. 

To overcome this limitation, this study proposes a data-driven approach to predict the propulsion power by learning data obtained from sensors installed on board the vessel. In this study, support vector regression (SVR) which shows excellent performance in prediction and pattern recognition is used to learn from data. For the illustration, the data obtained from an actual bulk is used to predict its propulsion power at various speeds and dynamic operational conditions. The prediction accuracy was also compared with that of ISO15016. The result shows that if the operational data are obtainable, the proposed model outperforms that of ISO15016.

The remainder of the paper is as follows. Section 2 briefly introduces the traditional methods that have been used to predict vessel propulsion power. Section 3 explains data that are used to train the prediction model. Section 4 explains the model development procedure in detail. Section 5 discusses the prediction result and compare them with other methods. Finally, Section 6 presents the conclusion and future works. 

## 2. Related Works

Predicting the propulsion power of a vessel at various operating conditions is considered a difficult problem because horsepower can be affected by several external factors such as wind, waves, and hull fouling. Those external factors would increase the vessel resistance, the force working against the vessel movement, requiring more propulsion power to maintain a certain vessel speed. Thus, several methodologies have analyzed the added resistance and speed loss to accurately predict the propulsion power.

As previously noted, the conventional method estimates the added resistance of the vessel, and uses this value in prediction of the required propulsion power [15]. The standard model for measuring the total resistance of the vessel is determined by the following equation:

(1) $$R_{total}=R_{friction}+R_{residual}+R_{wind}$$

where $R_{friction}$, $R_{residual}$ and $R_{wind}$ are resistance induced by the friction of the hull, residual resistance, and wind, respectively. $R_{friction}$ can be calculated by the following:

(2) $$R_{friction}=R_{hull}+R_{fouling}+R_{draft}$$

where $R_{hull}$ is the size of the hull’s wetted area, $R_{fouling}$ is fouling of the hull and $R_{draft}$ is specific frictional resistance coefficient. The residual resistance $R_{residual}$ is determined by the following:

(3) $$R_{residual}=R_{wave}+R_{eddy}$$

where $R_{wave}$ is the energy loss caused by waves created by the vessel during its propulsion through the water, while $R_{eddy}$ is eddy resistance which refers to the loss caused by flow separation which creates eddies, particularly at the aft end of the ship. In calm weather, $R_{wind}$ is proportional to the square of the ship’s speed, and proportional to the cross-sectional area of the ship above the waterline. Air resistance normally represents about 2% of the total resistance. Finally, the efficient propulsion power (*EHP*) of the vessel at a certain speed is determined by the following:

(4) $$EHP=SPEED\cdot R_{total}$$

where the *SPEED* is a specific vessel speed and $R_{total}$ is total resistance obtained from the above equation. 

In ISO15016, several procedures are introduced to correct added resistance [12]. For example, the added resistance due to the effect of short crested irregular wave, $R_{aw}$ can be calculated by the following:

(5) $$R_{aw}=2\int_{0}^{2\pi }\int_{0}^{\infty }\frac{R_{wave}(\omega ,\alpha ;V_{S})}{\zeta_{A}^{2}}E(\omega ,\alpha )d\omega d\alpha$$

where $\zeta_{A}$ is wave amplitude, ω is circular frequency of regular wave, α is angle between ship heading and incident regular wave, V_S_ is the vessel speed through the water, and E is directional spectrum. In calculation of $R_{aw}$, several methods such as the STAWAVE-1, STAWAVE-2, theoretical methods or the Seakeeping model test were proposed in [12]. However, such methods proposed in ISO15016 may not be appropriate to predict the propulsion performance in the actual operating condition because it requires data from the speed trial environment and requires too many input parameters. Even if such big data from operational conditions are available, it would take too much time and cost because the analysis procedure consists of complex equations which are computationally expensive.

Alternatively, Holtrop and Mennen’s method [16,17,18] is also widely used to estimate the resistance and propulsion power requirement during the vessel design phase. It is an empirical method that utilizes data accumulated from many model tests results. The model coefficient and equation are obtained from statistical analysis and regression applied to the data. Although this method requires less computation and numerical analysis, it only provides rough approximation of the resistance and propulsion power which may result in a less accurate result. Moreover, since data is obtained from the model experiments, prediction result cannot be applied to the actual operational condition. 

Another stream of works utilizes computational fluid dynamics (CFD) in the analysis of the vessel resistance and the propulsion performance [19,20,21]. CFD is a branch of fluid mechanics that uses numerical analysis and data structures to analyze and solve problems that involve fluid flows. In the vessel design phase, CFD have been utilized to predict the resistance by simulating the fluid performance of the designed hull form. Although a CFD-based model can accurately predict the resistance, the calculation of the fluid performance is computationally expensive, and usually requires several hours to complete. Thus, it is difficult to use CFD to predict the propulsion power at various operating conditions. To overcome the limitation of the above methodologies, this study proposes using machine-learning technique in the prediction of propulsion power using the data obtained from the actual operating environment. 

## 3. Data Collection 

**Figure 1:**
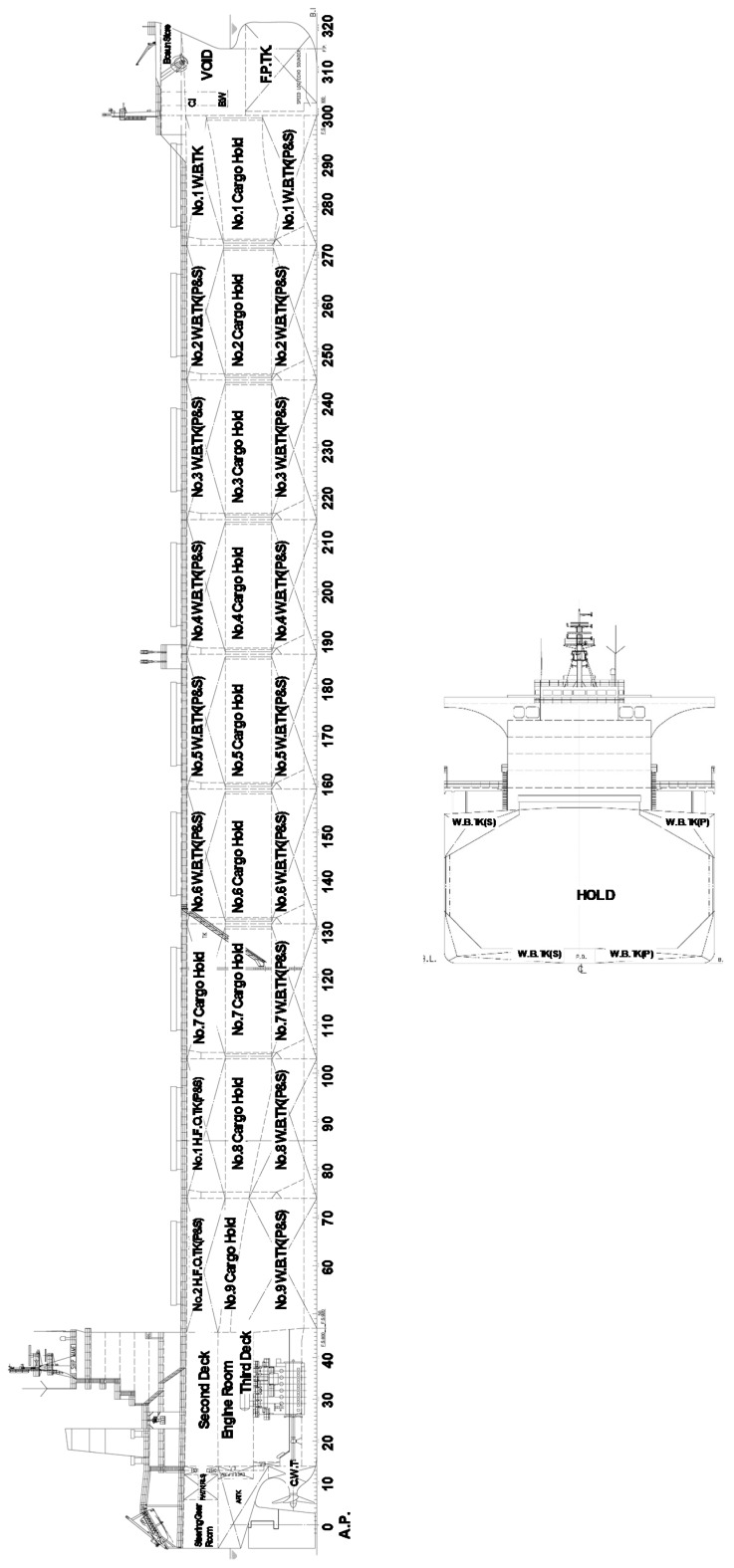
General arrangement of the vessel.

To predict the propulsion power, a 200,000-ton bulk cargo ship was chosen. The general arrangement of our target vessel is shown in Figure 1. Also, the detailed specification is depicted in Table 1. The target ship is dedicated to iron ore transportation and operates only on two regular routes: from South Korea to the US and from South Korea to Australia. Since it operates on a small number of stable routes, the data quality was thought to be reliable. For the propulsion system of the target ship, a fixed-pitch propeller whose pitch angle cannot be changed is adopted. 

**Figure 2:**
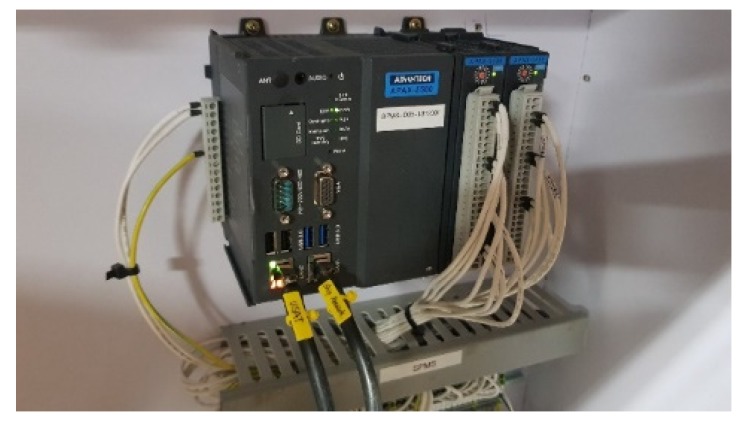
Data collection system in Voyage Data Recorder (VDR).

The detailed description of the dataset is shown in Table 2. Most of the data were collected using sensors installed onboard the vessel. The target feature to predict is shaft horsepower which indicates the amount of mechanical power that is delivered by the engine to the propeller shaft. For the input features, velocity, draft, rotation per minute of shaft (RPM), sea depth, tide, wave height, and wind vectors are chosen. Sensor information of each data feature is also illustrated in the same table. The collected data were then processed through the Voyage Data Recorder (VDR) system which digitizes, compress and stores the information in an externally mounted protective unit. Under regulations of the IMO (International Maritime Organization), a cargo ship that weighs more than 20,000 tons should be equipped with VDR. Although the primary purpose of the VDR is to assist marine causality investigation, the recorded data also can be used for performance monitoring of the vessel. The picture of the VDR system installed on the vessel is shown in Figure 2.

In processing the raw data obtained from different sensors, many computational steps would be required. For example, the shaft horsepower can be measured with the torque which can be measured with strain. The loading torque *T* is calculated by the following:

(6) $$T=\frac{\varepsilon \pi GD^{3}}{8}$$

where ε is shaft strain, G is shaft material shear modulus (pa) and D is shaft diameter (m). To accurately measure the torque, a strain gauge is attached to the propeller shaft of the vessel. The strain gauge measures the change in electrical resistance of the shaft and converts it into the torque. Several commercial system providers [22,23] as well as software vendors [24] are available for shaft power measurement. For our target ship, the SpecsVision-TPM system [25] is used for measuring and processing the shaft power. 

In addition to the shaft power measure, the raw data collected from the different sensors were processed by the data acquisition unit (DAU) of the VDR system. DAU transforms each sensor data according to the industrial standard protocol and synthesizes the data from different sensors into a structured data table wherein each datum is recorded at the same time periods. As a result, the synthesized data are transmitted throughout the satellite-based communication system and stored into the cloud database. As a result, each data observation is generated every 10 s, and overall 178,000 observations were collected over seven months (from 2016.01 to 2016.07). The data protocol information used to process the sensor data is also shown in Table 2. 

To collect the wave height, which cannot be measured directly on board the vessel, the National Oceanic and Atmospheric Administration (NOAA) database is utilized. NOAA collects spectral wave data using accelerometers on board the buoys which measure the heave acceleration of the buoy. A fast Fourier transform is then applied to obtain the wave height. For more detailed information about wave height measure of the NOAA database, the reader is referred to [26]. 

## 4. Development of Propulsion Power Prediction Model

### 4.1. Support Vector Regression (SVR)

As a machine learning model to predict the propulsion power of the vessel, SVR is applied. SVR, which was introduced by Drucker et al. [27], is an extension of support vector machine (SVM) that considers a regression problem as well. Like SVM, SVR tries to determine the hyperplane that maximizes the margin while ensuring that the error is tolerated. 

Suppose that a training data $\left\{(x_{1},y_{1}),(x_{2},y_{2}),\ldots ,(x_{n},y_{n})\right\}$ is given, SVR assumes the relationship between x and *y* represented by the following:

(7) $$f(x)=w^{T}\varphi (x)+b$$

where φ(x) denotes a kernel function that transforms the data into a higher dimensional space to make it possible to perform the linear separation, w is weight vector associated with vector x and b is coefficient. To find good estimator, SVR tries to find linear function that is as flat as possible. This can be formulated as a convex optimization problem and formulated as follows:

(8) $$J(w)=\frac{1}{2}w^{T}w$$

subject to $\forall n:\left|y_{n}-f(x_{n})\right|\leq \varepsilon$.

As shown in the above equation, SVR tries to minimize the norm of w while ensuring all residuals having a value less than ε. However, functions f(x) may not be exist for satisfying the above condition. To deal with such an infeasible constraint, we slightly modify the problem as follows:

(9) $$J(w)=\frac{1}{2}{\left\|w\right\|}^{2}+C\sum_{i=1}^{N}\left(\xi +\xi^{*}\right)$$

subject to:

$$\begin{array}{l} \forall i:y_{i}-J(w)\leq \varepsilon +\xi_{i}\\ \forall i:J(w)-y_{i}\leq \varepsilon +\xi_{{}_{i}}^{*}\\ \forall i:\xi_{i}\geq 0\\ \forall i:\xi_{i}^{*}\geq 0. \end{array}$$

The slack variables $\xi_{i}$ and $\xi_{i}^{*}$ serves as soft margin that allows the regression error of each data point i to exist up value $\xi_{i}$ and $\xi_{i}^{*}$. The constraint *C* controls the overfitting of the model by imposing a penalty on observations that lie outside the margin ε.

The solution of Equation (7) can be optimized by solving a dual problem that is computed as follows:

(10) $$L(a)=\frac{1}{2}\sum_{i=1}^{n}\sum_{j=1}^{n}(a_{i}-a_{i}^{*})(a_{j}-a_{j}^{*})K(x_{i},x_{j})+\varepsilon \sum_{i=1}^{n}(a_{i}+a_{i}^{*})-\sum_{i=1}^{n}y_{i}(a_{i}+a_{i}^{*})$$

subject to:

$$\begin{array}{l} \sum_{i=1}^{n}(a_{i}-a_{i}^{*})=0\\ \forall n:0\leq a_{i}\leq C\\ \forall n:0\leq a_{i}\leq C \end{array}$$

The function $K(x_{i},x_{j})=<\varphi (x_{i}),\varphi (x_{j})>$ is called as kernel function where $\varphi (x_{i})$ is a transformation that maps $x_{i}$ to a high-dimensional space. If we assume a linear model, linear kernel function $K(x_{i},x_{j})=x_{i}^{T}x_{j}$ is utilized. If the non-linear relationship is assumed, radial basis function (RBF) kernel $K(x_{i},x_{j})=\exp(-{\left\|x_{i}-x_{j}\right\|}^{2})$ or sigmoid kernel $K(x_{i},x_{j})=\tanh(\gamma (x_{i}^{T}x_{j})+\theta )$ are widely adopted. Now the function that is used to predict new value $x_{n}$ is equal to Equation (9):

(11) $$f(x)=\sum_{i=1}^{n}(a_{n}-a_{n}^{*})K(x_{n},x)+b$$

The predictive performance of SVR is significantly affected by the hyper-parameters. If we assume RBF as the kernel function, the performance of SVR is affected by hyper-parameters *C*, ε and σ. Parameters ε and *C* affect the way of penalizing the training error. The parameter ε controls the amount of error allowed in the model. If the residual is larger than ε, then the training error is penalized by *C*. Thus, too small *C* values may result in the overfitting, while too large *C* values may result in the underfitting of the model. On the other hand, the parameter σ determines the correlation of the kernel function. Thus, an optimal set of hyper-parameters among its possible combinations should be found. For a more detailed description of SVR, the reader is referred to [28]. 

### 4.2. Data Preprocessing

Before applying SVR, several preprocessing tasks were performed. In this step, data records that may lower the prediction performance were omitted from the dataset. Firstly, the observations with zero speed were omitted because it means that the vessel does not use propulsion power at all. Moreover, the observations whose vessel speed is less than 6 knots were also omitted because this indicates that the vessel is near a harbor and is likely to undergo frequent course changes. Such a situation may not be adequate for the propulsion power analysis. 

**Figure 3:**
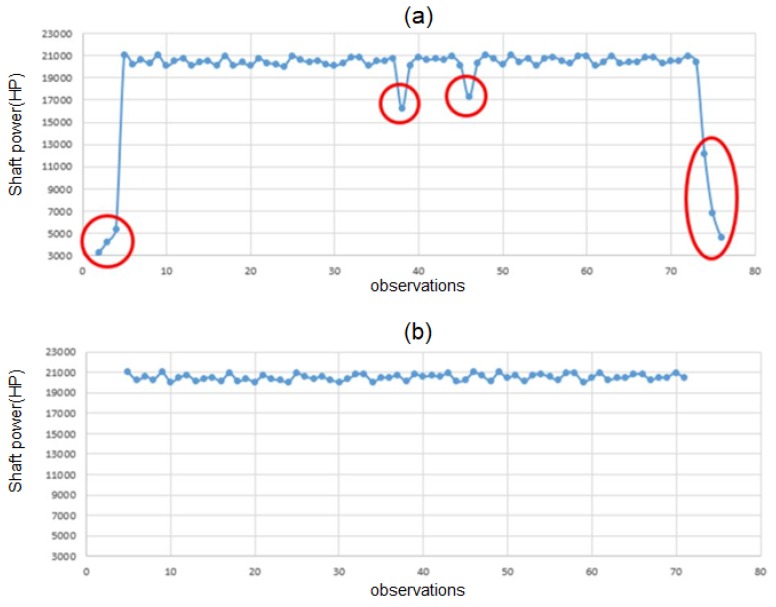
An example of outlier detection using Chauvenent’s criterion; 75 observations were randomly chosen from the dataset. (**a**) Before outlier detection. (**b**) After outlier detection.

Also, some outlier data that has abnormal shaft power value (output feature) is omitted from the dataset. To systematically determine the outlier, Chauvenet’s criterion, is utilized [29]. Given *N* samples of a dataset, an observation is considered as an outlier if *NP*(>*|z|)* < 0.5 where *P(*>*|z|)* is the cumulative probability of the observation is being more than *z* standard deviation of the mean. An example of outlier detection is shown in Figure 3. As shown in this figure, the outlier that shows a large deviation from the normal shaft power value is omitted from the dataset. 

Finally, feature scaling is applied to the dataset. Feature scaling is important for SVR, since it tries to maximize the distance between the separating plane and the support vectors. If one feature has larger magnitudes than others, it will dominate the other features when calculating the distance. In this study, we normalize each feature between the −1 to +1 intervals. All the preprocessing procedure was conducted using Pandas module with a python 3.6 environment.

### 4.3. Model Learning

The SVR algorithm was applied to the training dataset. In this study, RBF was chosen for the kernel function. The RBF is a widely adopted kernel function because the number of hyper parameters is small compared to that of other models without losing too much prediction performance. 

As previously mentioned, finding the optimal parameter is a crucial step for learning the SVR model. For the SVR model with RBF kernel function, three hyper-parameters *C*, ε and σ are required. In this study, to find the optimal hyper-parameter set, every possible parameter combination was validated by a K-fold cross validation approach. As a result, the optimal hyper parameter for the training dataset was C = 4950, σ= 0.6 and ε= 1.0. The experiment was conducted with Scikit-learn which is a famous open-sourced machine-learning library of python. 

## 5. Result

To validate our model, we divided the data into training and testing datasets. The data collected during first five months was used to train the model while the remaining two months were used to test the model. To measure the predictive performance of the model, RMSE (residual mean squared error) and R2-score were used. The evaluation result is shown in Table 3. The R2 score is 89.78% which indicates a fairly good performance for the regression.

**Figure 4:**
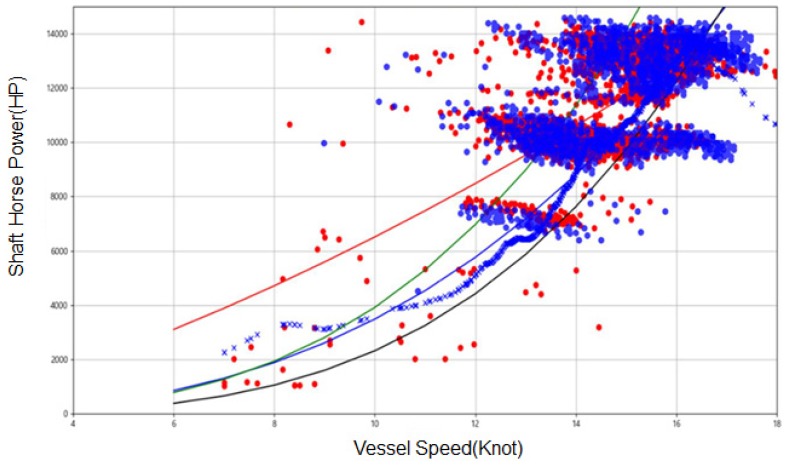
Comparison between predicted propulsion power vs. actual data.

In Figure 4, actual propulsion power vs. speed record is compared with the dataset predicted by our SVR model. Each red dot is actual propulsion power in the testing data set given at specific speed of the vessel. As shown in this figure, given the same speed level, the propulsion power shows large deviation. This deviation can be explained by the impact of external factors (wind, wave and tide). The blue dots are the data predicted by the SVR model. As shown in this figure, the SVR model shows almost the same pattern as actual data, which indicates that our SVR is a reliable tool to predict the propulsion power of the vessel.

**Figure 5:**
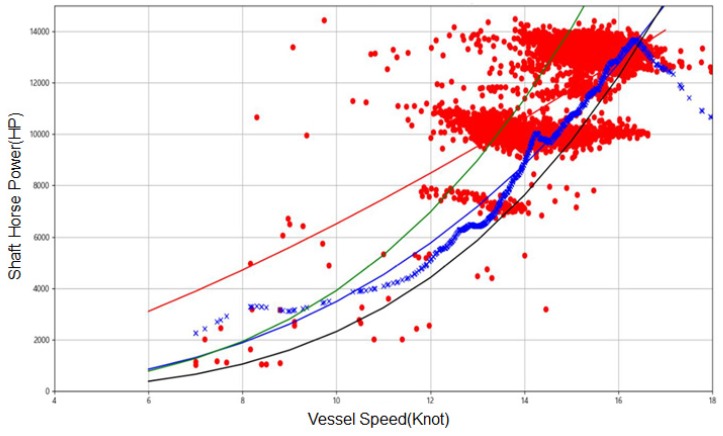
Predicted propulsion power without external effect.

Figure 5 shows the relationship between the speed and the propulsion power of the vessel. The green and black lines are estimated speed vs. power curve obtained from ISO15016 method. The green is obtained when the vessel is loaded with the cargo while the black line is obtained when the vessel is empty. The blue cross dots are speed vs. power relationship predicted by the SVR model while eliminating the impact of external factors. As shown in this figure, the predicted propulsion performance lies between green and black lines, which indicates that SVR model shows generic S/P relationship when it is not affected by the external factors (wind, wave, and tide). The SVR model shows an abnormal pattern when the input is over 18 notes because the data measured with vessel speed being over 18 notes is not available. 

We also have compared the prediction performance of the proposed model with that of an ISO15016 method. In order to do this, we have compared the prediction results of the two methods using the same testing dataset (2016.06.01~2016.08.01). When applying ISO15016 [12], the wind measure is adjusted based on the Fujiwara method (p.41) and the wave feature is adjusted based on the STAWAVE-2 method (p.45). STAWAVE-2 approximates the $R_{aw}$ in Equation (5) by the following:

(12) $$\begin{array}{l} R_{wave}=R_{AWML}+R_{AWRL}\\ R_{AWML}=4\rho sg\varsigma_{A}^{2}\frac{B^{2}}{L_{PP}}\bar{r_{aw}}(\omega )\\ R_{AWRL}=\frac{1}{2}\rho sg\varsigma_{A}^{2}B\alpha_{1}(\omega ) \end{array}$$

where $R_{AWML}$ and $R_{AWRL}$ is mean resistance increase due to wave motion and reflection respectively. $\bar{r_{aw}}(\omega )$ and $\alpha_{1}(\omega )$ are functions of circular frequency of regular wave ω which are explained in detail in [12]. For a more detailed procedure of STAWAVE-2, the reader is referred to [12].

The comparison result is shown in Table 4. As shown in the table, the SVR-based model outperforms the ISO15016 in both R2 score and RMSE. The ISO15016 method underperforms the SVR-based model especially when the vessel is in bad weather conditions.

## 6. Conclusions and Future Works

This study proposes a data-driven approach to predict propulsion power. Although several prediction methods are available, most of them are computationally expensive, and suffer from low prediction accuracy. Instead, we propose to use a machine-learning model that utilizes actual operational data of the vessel. In this study, support vector regression (SVR) is used to learn from 178,000 onboard data observations obtained from a bulk carrier that operates on solid lines. Compared to the conventional methods, the proposed model does not require complex equations and showed superior performance if only the big data of the solid line are secured. 

There are, however, further issues to explore to improve the model performance. Currently, the data feature related to the vessel status such as the ship damage, roughness of the hull, or engine performance degradation are not addressed. Accommodating such additional features may improve the model performance. Another issue is to compare numerous competing machine-learning algorithms and find the best models.

## Figures and Tables

**Table 1:** Specification of the target vessel.

Specification	
Length Overall	269.36 m
Length betw. perpendiculars	259.00 m
Breadth	43.00 m
Depth	23.80 m
Draught	17.3 m
Deadweight	152.517 metric t

**Table 2:** Data feature description.

Type	Feature Name	Description (unit)	Source	Sensors (Sensing Methods/Protocol)
Input Features	Ship Velocity	Velocity of the ship measured with Differential GPS. (knot)	Onboard Sensors	GPS Sensor (Position, date, time using GPS/NMEA0183)
	Draft	Vertical distance between waterline and the bottom of the hull. Draft is mainly affected by the weight of the cargo on board. (m)	Onboard Sensors	Draft Sensor (using Hydrostatic Level Pressure Transmitters/NMEA0183)
	RPM	Rotation per minute of shaft (RPM)	Onboard Sensors	RPM Indicator
	Sea Depth	Sea depth below the ship. Sea depth is measured with depth log recorded in VDR. (m)	Onboard Sensors	Echo Sounder (sonar wave is used to measure the time interval between emission and return of a pulse/NMEA0183)
	Tide	Tide around ship. Measured with the difference between speed through the water (STW) and speed over the ground (SOG). (m/s)	Onboard Sensors	Doppler log (using ultrasound and applying the Doppler effect to measures the speed of surface ship through water/NMEA0183)
	Wave Height	Height of wave.	NOAA database	Indirectly measured from heave acceleration of the buoys. (m)
	Wind Vector	A vector measure that indicates the speed and direction of the wind around the ship. (m/s)	Onboard Sensors	Anemometer (NMEA0183)
Output Features	Propulsion Power	Shaft horsepower of the ship. (hp)	Onboard Sensors	Shaft Torque Sensor (using strain gauge that converts torque into a change in electrical resistance/MODBUS)

**Table 3:** Prediction result.

Training dataset	2016.01.01 ~2016.05.31
Testing dataset	2016.06.01~2016.07.30
Kernel Function	RBF kernel
Hyper parameter	C = 4950, r = 0.6, epsilon = 1.0
R2 score	89.78%
RMSE	54 kW
Entry 2	data

**Table 4:** Comparison with ISO15016 method.

Date	2016.06.01 ~ 2016.07.30
Wind Correction	Fujiwara method
Wave Adjustment	STAWAVE-2
R2 score	30.23%
RMSE	985 kW

## References

[1] UNCTAD Review of Maritime Transport 2014. https://unctad.org/en/PublicationsLibrary/rmt2014_en.pdf.

[2] Journee J.M., Meijers J. (1980). Ship Routing for Optimal Performance.

[3] Chang Y.T., Song Y., Roh Y. (2013). Assessing Greenhous Gas Emissions from Port Vessel Operations at the Port of Incheon. Transp. Res. Part D Transp. Environ..

[4] Tilling F. (2017). A generic energy systems model for efficient ship design and operation. Proc. Inst. Mech. Eng. Part M J. Eng. Marit. Environ..

[5] Papanikolaou A. (2010). Holistic ship design optimization. Comput.-Aided Des..

[6] Calcagni D., Bernardini G., Salvatore F. Automated marine propeller optimal design combining hydrodynamics models and neural networks. Proceedings of the 11th International Conference on Computer Applications and Information Technology in the Maritime Industries.

[7] Hellstrom T. (2004). Optimal Pitch, Speed and Fuel Control at Sea. J. Mar. Sci. Technol..

[8] Lin Y.H., Fang M.C., Yeung R.W. (2013). The optimization of ship weather-routing algorithm based on the composite influence of multi-dynamic elements. Appl. Ocean Res..

[9] van den Boom H.J., Hasselaar T.W. (2014). Ship speed-power performance assessment. Transactions—The Society of Naval Architects and Marine Engineers.

[10] van den BOOM H.J., Huisman H., Mennen F. (2013). New Guidelines for Speed/Power Trials: Level Playing Field Established for IMO EEDI.

[11] Arribas F.P. (2007). Some methods to obtain the added resistance of a ship advancing in waves. Ocean Eng..

[12] ISO15016 Ships and Marine Technology—Guidelines for the Assessment of Speed and Power Performance by Analysis of Speed Trial Data. https://www.iso.org/standard/61902.html.

[13] Insel M. (2008). Uncertainty in the analysis of speed and powering trials. Ocean Eng..

[14] IMO Guidelines for Voluntary Use of the Ship Energy Efficiency Operational Indicator (EEOI). http://www.imo.org/en/OurWork/Environment/PollutionPrevention/AirPollution/Pages/Technical-and-Operational-Measures.aspx.

[15] MAN Diesel & Turbo (2011). Basic Principles of Ship Propulsion.

[16] Holtrop J. (1984). A statistical re-analysis of resistance and propulsion data. Int. Shipbuild. Prog..

[17] Holtrop J., Mennen G.G.J. (1978). A statistical power prediction method. Int. Shipbuild. Prog..

[18] Holtrop J., Mennen G.G.J. (1982). An approximate power prediction method. Int. Shipbuild. Prog..

[19] El Moctar B., Kaufmann J., Ley J., Oberhagemann J., Shigunov V., Zorn T. Prediction of ship resistance and ship motions using RANSE. Proceedings of the Gothenburg—A Workshop on Numerical Ship Hydrodynamics.

[20] Simonsen C.D., Otzen J.F., Joncquez S., Stern F. (2013). EFD and CFD for KCS heaving and pitching in regular head waves. J. Mar. Sci. Technol..

[21] Tezdogan T., Demirel Y.K., Kellett P., Khorasanchi M., Incecik A., Turan O. (2015). Full-scale unsteady RANS CFD simulations of ship behaviour and performance in head seas due to slow steaming. Ocean Eng..

[22] Datum electronics Marine Shaft Power Meter Product Overview. https://www.datum-electronics.co.uk.

[23] Kyma A.S. Kyma Test Power Meter. https://kyma.no/wp-content/uploads/2019/03/TPM-Kyma-test-power-meter-brochure.pdf.

[24] Keysight Technologies Data Acquisition Control & Analysis. https://www.keysight.com/us/en/software/application-sw/benchvue-software/data-acquisition-control---analysis.html.

[25] Specs Specvision. http://www.specs.co.kr/eng/spmseemspage_specsvision_1.htm..

[26] NOAA (National Oceanic and Atmospheric Administration) How Are Spectral Wave Data Derive from buoy Measurements?. https://www.ndbc.noaa.gov/wave.shtml.

[27] Drucker H., Burges C.J., Kaufman L., Smola A.J., Vapnik V. (1997). Support vector regression machines. Advances in Neural Information Processing Systems.

[28] Smola A.J., Schölkopf B. (2004). A tutorial on support vector regression. Stat. Comput..

[29] Ross S.M. (2013). Peirce’s criterion for the elimination of suspect experimental data. J. Eng. Technol..

